# Varifocal Metalens Using Tunable and Ultralow‐loss Dielectrics

**DOI:** 10.1002/advs.202204899

**Published:** 2023-01-03

**Authors:** Mengyun Wang, June Sang Lee, Samarth Aggarwal, Nikolaos Farmakidis, Yuhan He, Tangsheng Cheng, Harish Bhaskaran

**Affiliations:** ^1^ Department of Materials University of Oxford Oxford OX1 3PH UK

**Keywords:** near‐IR wavelengths, Sb_2_Se_3_, tunable metalens, ultralow‐loss phase‐change materials

## Abstract

The field of flat optics that uses nanostructured, so‐called metasurfaces, has seen remarkable progress over the last decade. Chalcogenide phase‐change materials (PCMs) offer a promising platform for realizing reconfigurable metasurfaces, as their optical properties can be reversibly tuned. Yet, demonstrations of phase‐change metalenses to date have employed material compositions such as Ge_2_Sb_2_Te_5_, which show high absorption in the visible to near‐IR wavelengths particularly in their crystalline state, limiting the applicability. Here, by using a low‐loss PCM Sb_2_Se_3_, for the first time, active polarization‐insensitive phase‐change metalenses at near‐IR wavelengths with comparable efficiencies in both material states are shown. An active metalens with a tunable focusing intensity of 95% and a focusing efficiency of 23% is demonstrated. A varifocal metalens is then demonstrated with a tunable focal length from 41 to 123 µm with comparable focusing efficiency (5.7% and 3%). The ultralow‐loss nature of the material introduces exciting new possibilities for optical communications, multi‐depth imaging, beam steering, optical routing, and holography.

## Graphical abstract

Sb_2_Se_3_ is a remarkable, highly efficient (*κ* = 0), and tunable (∆*n* = 0.75) dielectric material at near‐IR wavelengths. This ultralow‐loss material is utilized to realize reconfigurable metalenses at near‐IR. Taking advantage of the low‐loss nature of the material, both single focal metalens with tunable focusing intensity as well as varifocal metalens with comparable efficiencies are demonstrated.

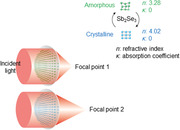

## 1 Introduction

Subwavelength metasurfaces have sparked a great deal of interest, as they can manipulate the wavefronts of light for compact adaptive optics in applications such as light detection and ranging systems, dynamic holography, artificial‐intelligence using robotic eyes, and self‐driving vehicles, amongst other applications.^[^ ^1^ ^]^ The ability to efficiently reconfigure such metasurfaces at‐will is the current goal, as that will enable such flat lenses to also have variable focusing capability, achieving the true promise of such systems. A variety of approaches have been investigated, utilizing mechanical actuation methods, electrically‐ or thermally‐induced volatile modulations in refractive index and recently tunable materials,^[^ ^2^ ^]^ among which, phase change materials are one of the most promising approaches. Started with the work by Zheludev and colleagues,^[^ ^3^ ^]^ chalcogenide phase‐change material (PCM)‐based metasurfaces have become a rapidly growing field of research in recent years, and hold great potential for applications in tunable photonics due to their significant advantages in terms of speed, power, and design flexibility.^[^ ^4^ ^]^

Most demonstrations of phase change metasurfaces use the phase change material, Ge_2_Sb_2_Te_5_ (GST).^[^ ^4^, ^5^ ^]^ However, the high absorption loss of crystalline‐state GST lowers the efficiency of the devices, limiting their performance and applicability; this has led to significant research into low‐loss phase change materials. Recently, Ge_2_Sb_2_Se_4_Te_1_ (GSST), which offers exceptionally broadband transparency in the infrared spectral regime for both its amorphous and crystalline phases has been demonstrated for reconfigurable metasurfaces, with realizations of reconfigurable beam steering at 1550 nm^[^ ^5c^ ^]^ and tunable bifocal metalens operating at 5.2 µm.^[^ ^6^ ^]^ Antimony triselenide (Sb_2_Se_3_) has also been suggested as an ultralow‐loss chalcogenide PCM for photonics,^[^ ^7^ ^]^ enhanced meta‐displays,^[^ ^8^ ^]^ and active metalenses.^[^ ^9^ ^]^ However, no experimental demonstration of their use in tunable metalenses has been reported thus far. In this paper, we experimentally demonstrate metalenses consisting of high‐index and low‐loss Sb_2_Se_3_ nanostructures, exhibiting excellent focusing tunability.

**Figure 1:**
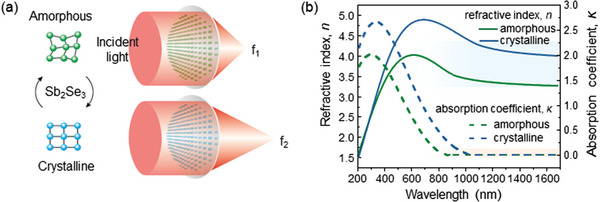
a) Schematic of the tunable metalens. Incident light is focused on the first focal plane (f_1_) when the Sb_2_Se_3_ is in the amorphous state and is focused at the second focal plane (f_2_) in the crystalline‐state Sb_2_Se_3_, respectively. b) The measured complex refractive index of ultralow‐loss Sb_2_Se_3_.

As shown in **Figure** **1**, we demonstrate the use of Sb_2_Se_3_ in tunable metalenses; both amorphous and crystalline states of Sb_2_Se_3_ exhibit a large real part (n) and negligible imaginary part (*κ*) of the refractive index, with a significant change in the real part (∆*n*) in the near‐IR range (Figure 1b). These properties satisfy the conditions for sustaining tunable Mie‐type resonances to realize a Huygens’ metasurface.^[^ ^10^ ^]^ By using this concept, we demonstrate a single‐focal metalens with diffraction‐limited focusing performance and a focusing efficiency of 23%. The focusing intensity can be tuned upon transition to the crystalline phase of the Sb_2_Se_3_ with 95% intensity modulation. We then demonstrate a varifocal metalens whose focal length is modulated from 41 µm (amorphous‐state Sb_2_Se_3_) to 123 µm (crystalline‐state Sb_2_Se_3_), while maintaining comparable focusing efficiencies.

## 2 Results and Discussion

### 2.1 Design and Simulation of Sb_2_Se_3_ Nanopillars

**Figure 2:**
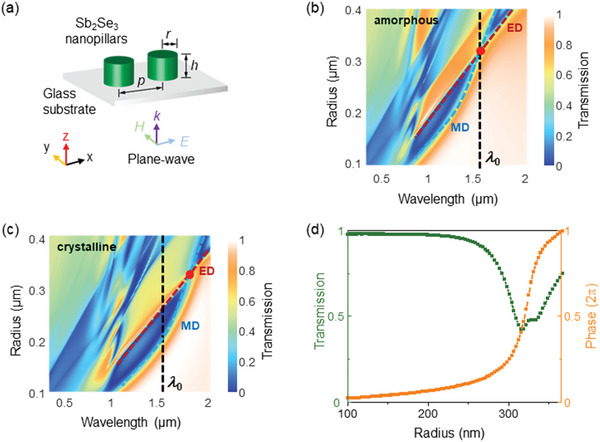
a) Sketch of the Sb_2_Se_3_ nanopillar arrays, showing their h, r, and p. The structure is illuminated by a plane wave propagating along *z*‐axis and linearly polarized parallel to the x‐axis. b) Simulated transmission spectra for amorphous‐state Sb_2_Se_3_ in terms of radius and wavelength. The resonance overlap (indicated by red dots) of ED (marked by red dash line) and MD (indicated by blue dash line) can be tuned to the working wavelength (*λ*_0_ = 1550 nm) by controlling the height and radius of Sb_2_Se_3_ nanopillars. The optimal height h = 278 nm, p = 3r. c) Simulated transmission spectra after the Sb_2_Se_3_ is switched to crystalline state, h = 278 nm. d) The transmission spectrum and phase response through amorphous‐state Sb_2_Se_3_ nanopillars with varying radii at the resonance‐overlapped wavelength (1550 nm), showing full 2*π* phase modulation and minimized transmission loss.

We employ the principle of Huygens’ metasurfaces for the design of Sb_2_Se_3_ nanopillars,^[^ ^11^ ^]^ which exploit the mode overlap between the electric and magnetic dipoles of Mie resonances, thereby realizing a 2*π* phase shift with minimized transmission loss. Such resonances are engineered by the optimization of Sb_2_Se_3_ cylindrical nanopillars assembled in a periodic array with designed height (h), radius (r), and period (p), as described in **Figure** **2**a. The symmetric configuration of the nanopillars enables polarization‐insensitive response of the metasurfaces. The calculated transmission spectra (Lumerical, FDTD) with varying radii (100 – 400 nm) of the nanopillars are shown in Figures 2b,c, where the spectral dips represent multipolar Mie resonances. One can find the spectral overlap between electric dipole (ED) and magnetic dipole (MD) resonances at 1550 nm (*λ*_0_) for the amorphous state of the material (Figure 2b); this overlap is redshifted when the material is crystallized (Figure 2c). Thus, at our target wavelength (1550 nm), the optimized Sb_2_Se_3_ nanopillars support both ED and MD resonances in their amorphous state, but not in the crystalline state. Such mode overlap allows destructive interferences of the incident and resonantly induced scattered waves, resulting in high transmission (with average transmission of 81.69%) and a full 2*π* phase shift as shown in Figure 2d. Upon crystallization, the ED and MD resonances are spectrally separated and the mode overlap at 1550 nm ceases to exist, resulting in low transmission and at‐most *π* phase‐shift at each resonance, as shown in Supporting information Figure S1. Moreover, the overall spectra can be blue shifted by decreasing the height of nanopillars (*h* = 225 nm) so that the mode overlap and a 2*π* phase control are achieved at the target wavelength in the crystalline state, but not in the amorphous state (Figure S2, Supporting Information). We use this property of the Sb_2_Se_3_ to realize tunable metalenses in the following sections.

### 2.2 Single Focal Metalenses with Intensity Tunability

**Figure 3:**
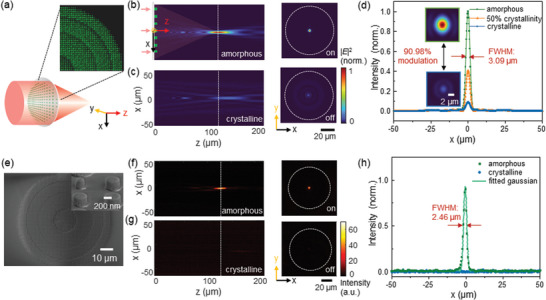
Intensity‐tunable single‐focal Sb_2_Se_3_ metalens. a) Schematic view of the single focal metalens design, with a quarter of the layout shown at the upper right. b) Left: the simulated electric filed intensity along the direction of light propagation (*z*‐axis). Right: the simulated electric field intensity at the focal plane. The white dotted line circles the dimensions of the metalens. c) The simulated electric filed intensity distributions when the Sb_2_Se_3_ is changed to crystalline state. d) Light intensity distributions along *x*‐axis at the focal point. e) The top‐view SEM of the fabricated metalens (inset: a 30°‐titled top‐view SEM of the nanopillars). f) The measured light intensity along the direction of light propagation (left) and at the focal plane (right) for amorphous Sb_2_Se_3_. g) The measured light intensity when the Sb_2_Se_3_ is crystallized. h) The measured light intensity distributions along *x*‐axis at the focal plane, which is normalized to the maximum intensity when light is focused.

First, we design a tunable single‐focal metalens consisting of periodically arranged Sb_2_Se_3_ nanopillars, which focuses light with a focal length of 120 µm when the Sb_2_Se_3_ is in the amorphous state, but does not focus light when in the crystalline state. The layout of one quarter of the designed metalens is shown in **Figure** **3**a, with a full‐lens radius R of 35 µm (corresponding to a numerical aperture N.A. value of 0.28). More information for the designing of metalens can be found in Figure S3 (Supporting Information). We calculate the 2D electric‐field distributions through the metalens in its amorphous state as shown in Figure 3b, where a focal spot is observed with a focal length of 116.63 µm. The slight aberration can be improved by optimizing the phase distributions^[^ ^9^, ^12^ ^]^ or increasing the feature sizes (i.e., N.A) of the metalens.^[^ ^13^ ^]^ The calculated full‐width at half‐maximum (FWHM) is 3.09 µm (approximating the theoretical diffraction limit ∼λ/2NA), with a focusing efficiency of 27.09%. The focusing efficiency is defined as the ratio of the light intensity concentrated at the focal spot (within a radius of three times the FWHM) over the transmitted power through the bare glass substrate without the metalens.^[^ ^6^, ^11^ ^]^ However, when the material is changed to crystalline state (Figure 3c), the focus is lost due to the mismatch of optical phase modulation (Figure S3c, Supporting Information). These simulations indicate that our near‐IR metalens can be focused on (amorphous) and off (crystalline) at a target focal plane in response to the phase of the Sb_2_Se_3_.

The contrast of the focusing intensity upon different crystalline phases of Sb_2_Se_3_ is simulated and described in Figure 3d, where the 1D normalized electric‐field intensity profile shows an intensity modulation (∆I/I_0_, I_0_ is the focusing light intensity when the Sb_2_Se_3_ is in amorphous state) of 90.98% at the focal point. Partial crystallization of Sb_2_Se_3_ (e.g., 50% crystallinity) further allows fine tuning of the focusing intensity. Similarly, a focusing‐intensity tunable metalens which focuses light for crystalline‐state Sb_2_Se_3_, but loses its focusing in the amorphous state can be achieved (Figure S4, Supporting Information). Such intensity modulation is not a consequence of the absorption loss of the material, but arises from the engineering of optical phase profiles. The high index and negligible loss of Sb_2_Se_3_ permits excellent lens performance for both amorphous and crystalline states of Sb_2_Se_3_. Figure S5 (Supporting Information) shows the optical response when light propagates through the optimized Sb_2_Se_3_ nanostructures, representing near‐zero absorption but only transmission and reflection. The non‐trivial reflection is still observed due to some spatial mode mismatch between electric‐ and magnetic‐dipoles. This limitation can be improved by either further optimizing nanopillar geometries^[^ ^14^ ^]^ or replacing the surrounding medium with higher index material such as transparent polymer (n = 1.4).^[^ ^11b^ ^]^ This enhances the field confinement, allowing the incident light to propagate through without backscattering (Figure S6, Supporting Information). However, further numerical optimization and design, e.g., local periodization or global optimization, will be required to minimize the interelement electromagnetic coupling which will improve the focusing efficiency of a Huygens’ metalens.^[^ ^15^ ^]^

We proceed to experimentally demonstrate such metalens. We fabricate the structures using electron‐beam lithography (EBL) in combination with reactive‐ion etching on a quartz‐coated glass substrate with sputtered Sb_2_Se_3_. Detailed fabrication procedures are described in experimental section and Figure S6a (Supporting Information). The scanning electron microscope image of our metalens is shown in Figure 3e, exhibiting close agreement with the layout in Figure 3a and nearly vertical sidewalls. We use the customized measurement setup (see experimental section and Figure S6b) to verify the focusing performance of our metalens. As shown Figure 3f–h, our metalens shows a well‐defined diffraction‐limited focal spot at 115.2 µm with a focusing efficiency of 23.0% and FWHM of 2.46 µm when the Sb_2_Se_3_ is in amorphous state. When the device was heated on a hotplate at 220 °C for 5 min to crystallize the material, the metalens shows negligible focusing at the same focal point (∆I/I_0_ = 94.8%) as shown in Figure 3g. The measured light intensity distributions are in excellent quantitative agreement with the calculated results and consistent between different devices, confirming the successful experimental demonstration of intensity tunable Sb_2_Se_3_ metalens. Besides, the focusing efficiency of 23.0% achieved here is mainly limited by the transmission.

### 2.3 Varifocal Metalens without Degrading Focusing Efficiency

**Figure 4:**
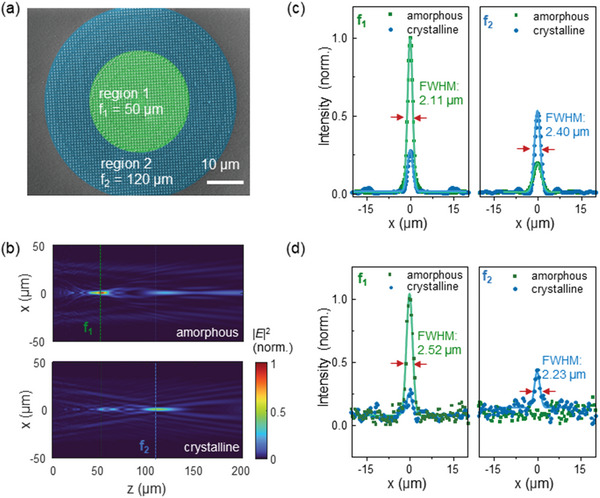
Characterizations of the varifocal metalens. a) The false colored SEM image of the fabricated varifocal metalens with region 1 (len radius R < 17.5 µm) focusing light to the first focal point f_1_ when the metalens is in the amorphous state, and region 2 (17.5 µm < R < 35 µm) focusing light at the second focal point f_2_ when it is in the crystalline state. b) The simulated far‐filed intensity along the direction of light propagation for amorphous (upper) and crystalline‐state of Sb_2_Se_3_ (bottom). The distances of the focal planes are z = 52.6 µm for f_1_ and z = 110.2 µm for f_2_. c) The simulated far‐field intensity at the focal planes for amorphous (green) and crystalline‐state (blue) of Sb_2_Se_3_. d) The measured light intensity distributions at the two focal planes f_1_ (left) and f_2_ (right), which are normalized to the maximum intensity when light is focused at first focal point f_1_.

We now extend the use of low‐loss Sb_2_Se_3_ to create a varifocal metalens that can tune its focal length by changing the crystalline state of the material. As shown in **Figure** **4**a, the metalens is designed with two regions concentric with each other; each region is designed for a different focal length (Figure S9, Supporting Information). The inner region (region 1) has a focal length of 50 µm (f_1_) when the Sb_2_Se_3_ is in the amorphous state; in the crystalline state, it will not focus light. On the other hand, the outer region (region 2), focuses light at 120 µm (f_2_,) when in the crystalline state, but does not focus light in its amorphous state. Thus we implement a single metalens that varies its focal length from 50 µm (f_1_) to 120 µm (f_2_).

Figures 4b,c shows the simulated results of electric‐field intensity which indicates light focusing at 52.6 µm (f_1_) when the material is in the amorphous state, and focusing at 110.2 µm (f_2_) when the Sb_2_Se_3_ is in the crystalline state. The calculated focusing efficiencies are comparable, which are 7.58% for f_1_ and 9.39% for f_2_, demonstrating the low‐loss nature of both states. The FWHM at each focal plane is 2.11 and 2.40 µm, respectively (Figure 4c), with a modulation of focusing intensity being 72.55% (at f_1_) and 61.96% (at f_2_) upon crystallization of the Sb_2_Se_3_ nanopillars. The decrease in focusing efficiency and modulation range of varifocal metalens, compared to the single focal metalens, is due to the shared‐aperture layout. Further improvements to enable binary switching of metasurfaces between arbitrary phase profiles without using shared‐aperture layout will increase performance, e.g., by using a more efficient design methodology to select optimal meta‐atom geometries.^[^ ^6^ ^]^ We then experimentally measure this on the fabricated sample (Figure 4a), and our measurements are shown in Figure 4d. The measured FWHM is 2.52 µm for f_1_ and 2.23 µm for f_2_. The calculated and measured light intensity distributions agree qualitatively with more information shown in Figure S7 (Supporting Information). The measured focal lengths are 41 µm for f_1_ (amorphous) and 123 µm for f_2_ (crystalline) with comparable focusing efficiency (5.74% and 2.97%, respectively). This is the first demonstration of a polarization‐insensitive varifocal metalens operating in the near‐IR using low‐loss PCMs.

Varifocal metalenses operating at visible and near‐IR wavelengths play an important role in imaging applications, and have been a long‐sought desirable feature for adaptive optics. Table S1 (Supporting Information) summarizes the state of the art of previously demonstrated varifocal metalenses using various tuning mechanisms. While mechanical actuations, e.g. using MEMS^[^ ^2e^ ^]^ and stretchable substrates^[^ ^11c^ ^]^ have been demonstrated as efficient approaches for designing high‐performance varifocal metalenses, they require cumbrous physical movements and have difficulties to miniaturize the system size. The integration of liquid crystals and static metasurfaces provides an opportunity to create compact tunable metalenses,^[^ ^16^ ^]^ but it relies on the manipulation of the light polarization to vary the focal planes and comes with polarization‐sensitive focusing, which is unwanted in imaging applications. Recently, PCM‐based varifocal metalenses have been demonstrated at wavelengths in the mid‐IR at 3^[^ ^17^ ^]^ and 5.2 µm,^[^ ^6^ ^]^ especially, the latter utilized a novel low‐loss PCM, GSST. GSST is a remarkable and highly efficient material for such use in the mid‐IR and our work fulfills a similar role in the important near‐IR wavelengths. As stated above, our metalens does not exhibit significant absorption loss, and comparable focusing efficiency can be achieved at both focal planes in response to the two states of Sb_2_Se_3_. Here, we show a focal length tuning from 41 to 123 µm, and our approach is generic and applicable to design varifocal metalenses switchable between arbitrary focal lengths as both amorphous and crystalline‐state Sb_2_Se_3_ can be used for high‐performance intensity‐tunable metalenses. The design can also be generalized to other novel high‐index and low‐loss PCMs in the future. Furthermore, optical switching^[^ ^7^ ^]^ and transparent on‐chip heaters^[^ ^18^ ^]^ for reversible switching of PCMs have also been validated in recent years. With future implementation of theses switching methods and our tunable metalens using low‐loss PCMs, we envision that it will enable rapid zooming and auto‐focusing function in the near‐IR region without involving bulky mechanical moving parts. This work sets the stage for ultralow‐loss Sb_2_Se_3_ to be utilized to design low‐loss and highly efficient switchable optical devices in near‐IR region, foreseeing great potential in next‐generation compact adaptive optical systems.

## 3 Conclusion

In summary, we have proposed and experimentally demonstrated tunable metalenses operating at near‐IR wavelengths utilizing the ultralow‐loss PCM Sb_2_Se_3_. By employing this low‐loss and high‐index PCM, a single all‐dielectric layer of Sb_2_Se_3_‐based nanopillars with relatively low aspect ratio (*h*/(2 × *r*_min_) = 1.35) is sufficient to support Mie‐type resonances of electric and magnetic modes, achieving full 2*π* optical phase control with minimization of the transmission loss. A single focal metalens with high focusing efficiency (23%) and excellent intensity tunability (95% intensity modulation range) is demonstrated. Furthermore, a varifocal metalens with diffraction‐limited performance and comparable focusing efficiency at both focal planes is demonstrated by arranging the lens area into two regions with each region focusing light at one focal point for each state of the material. This fulfills the long‐sought goal of realizing compact varifocal metalens at near‐IR wavelengths without decreasing its focusing efficiency. Our work presents the first demonstration of near‐IR varifocal metalenses utilizing ultralow‐loss PCMs, paving new possibilities for the designing of low‐loss and highly efficient switchable optical devices in the near‐IR range. Future improvements to maximize the transmission and integrate on‐chip heaters will enable high‐efficiency and dynamic tunable metalenses, which are of great potential to achieve miniaturized, low‐loss adaptive optics, and tunable photonic devices for versatile applications ranging from multi‐depth imaging, information storage, to dynamic displays, image encryption, and anti‐counterfeiting.

## 4 Experimental Section

### Modeling

Numerical simulations were carried out with a finite‐difference time‐domain simulations (Lumerical, FDTD) to calculate electric‐field distribution and transmissive spectral responses of the metalens, under plane‐wave illumination.^[^ ^19^ ^]^ For each unit cell of the metalens, a periodic array of nanopillars was used to calculate the spectral responses of transmitted intensity and phase as a function of nanopillar geometrical dimensions. Far‐field distribution of the electric‐field was calculated to characterize the focusing performance of the metalens. The dielectric function of the Sb_2_Se_3_ film was obtained from ellipsometry.

### Device Fabrication

The Sb_2_Se_3_ of 270 nm thickness was deposited onto a quartz‐coated glass substrate by radio‐frequency sputtering (AJA international). Standard EBL was performed on a positive e‐beam resist (CSAR) with a conductive charge dissipation layer (ESPACER 300Z) to define the nanopillars. After development, the patterned substrate was subjected to a lift‐off process followed by deposition of Cr of 10 nm thickness via thermal evaporation. Then, the sample was etched with using Cr as a hard mask by reactive ion etching (Oxford Instruments) with a gas mixture of CHF_3_ (44 sccm) and O_2_ (6 sccm).

### Optical Characterization

The optical measurement was conducted using a customized microscope system as shown in Figure S7 (Supporting Information). The sample was placed on a piezo‐motorized scanning stage (Physik Instrumente). The near‐IR (1570 nm) laser was (Keysight, N7711A) propagated onto the sample through a 0.28‐NA objective lens. The 2D light‐intensity images were recorded by a digital CCD camera (CamIR, Scintacor). The longitudinal light intensity distribution through the metalens was acquired through a stack of 2D images by scanning the metalens position along light propagation direction (*z*‐axis) with a step‐size of 200 nm. The stage movement and data acquisition were controlled by an open source program (µmanager).^[^ ^20^ ^]^ The focusing efficiency was normalized by the transmitted intensity through a bare glass substrate measured under the same setup. For the measurement of crystalline‐state Sb_2_Se_3_ metalens, the fabricated metalens was annealed at 220 °C for 5 min. The varifocal metalens required higher temperature and longer time to observe the presence of the second focal point (270 °C for 30 min).

## Conflict of Interest

The authors declare no conflict of interest.

## Author Contributions

M.W. and J.S.L. contributed equally to this work. All authors contributed substantially. M.W. and J.S.L. performed the numerical simulation, designed the experiment, and built the optical measurement setup. M.W. fabricated the samples. J.S.L. and M.W. carried out the optical measurements and analyzed the data. T.C. calibrated the deposition of the material. S.A., N.F., and Y.H. helped in the fabrication process. H.B., J.S.L., and M.W. conceived of the presented idea. H.B. supervised the experiment. All the authors discussed the results and prepared the manuscript.

## Supporting information

Supporting information

## Data Availability

The data that support the findings of this study are available from the corresponding author upon reasonable request.
