# Supplementary material for: Varifocal Metalens Using Tunable and Ultralow‐loss Dielectrics
Source: Adv Sci (Weinh). 2023 Jan 3;10(6):2204899. doi: 10.1002/advs.202204899 (PMC9951390; doi:10.1002/advs.202204899)
Supplement: Supplementary file 1 — Supporting information [file ADVS-10-2204899-s001.pdf]

ADVANCED SCIENCE
Open Access

Supporting Information

for *Adv. Sci.*, DOI 10.1002/advs.202204899

Varifocal Metalens Using Tunable and Ultralow-loss Dielectrics

*Mengyun Wang, June Sang Lee, Samarth Aggarwal, Nikolaos Farmakidis, Yuhan He, Tangsheng Cheng and Harish Bhaskaran**

Supporting information

Varifocal metalens using tunable and ultralow-loss dielectrics

*Mengyun Wang[1, †], June Sang Lee[1, †], Samarth Aggarwal[1], Nikolaos Farmakidis[1], Yuhan He[1,] Tangsheng Cheng[1] and Harish Bhaskaran[1, *]*

[1] Department of Materials, University of Oxford, Oxford, OX1 3PH, UK

[†]These authors contributed equally to this work.

[*] Corresponding author.

1. Transmission, phase response and electric and magnetic field profiles

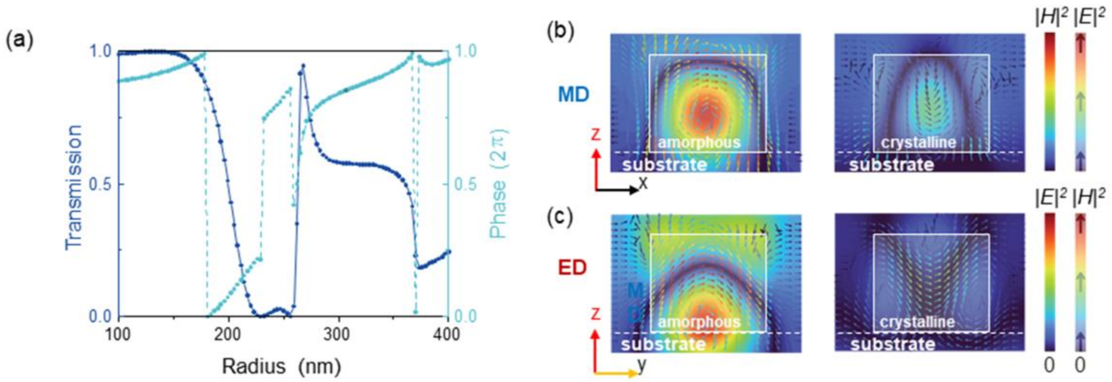

Figure S1 (a) The simulated transmission spectrum and phase response after the optimized Sb_2Se_3 nanopillars are switched to crystalline state. (b) Electric and magnetic field profiles (at xz-plane) of the MD resonance at the resonance-overlapping wavelength (1550 nm) for amorphous-state Sb_2Se_3 (left) and crystalline-state Sb_2Se_3 (right). (c) Electric and magnetic field distributions (at yz-plane) of the ED resonance for amorphous and crystalline-state Sb_2Se_3. The Sb_2Se_3 nanopillar is illuminated with a plane-wave light source propagating along z-axis and linearly polarized parallel to the x-axis.

The field distributions at 1550 nm for the optimized amorphous-state Sb_2Se_3 nanopillar (h=278 nm, r=320 nm, p=3r) reveal strong field enhancement from magnetic dipolar (MD) and electric dipolar (ED) resonances and the generated circulating electric field and magnetic field indicated by the colored arrows[1, 2].

2. Optimization of the crystalline-state Sb_2Se_3 nanopillars

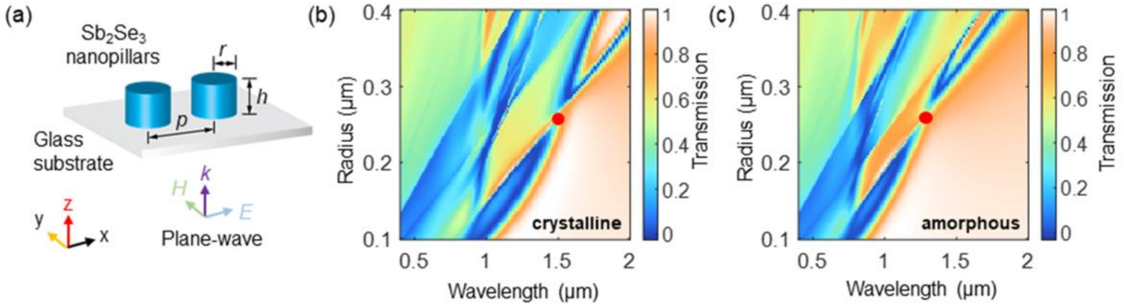

Figure S2. (a) Sketch of the periodic crystalline-state Sb_2Se_3 nanopillars, showing its height (*h*), radius (*r*), and period (*p*). (b) Simulated transmission spectra in terms of radius and wavelength. At the optimal height *h*= 225 nm for crystalline-state Sb_2Se_3, the resonance overlap of ED and MD (indicated by red dots) can be tuned to the working wavelength (λ=1550 nm). (c) Simulated transmission spectra when the Sb_2Se_3 is switched to amorphous state. The resonant overlapping wavelength is blue

3. Design of the metalens

Here explains the detail for the designing of a metalens with a radius *R* of 35 μm and focal length *f* of 120 μm. For design simplicity, the periodicity of the array of cylindrical Sb_2Se_3 nanopillars is fixed at 1020 nm. In this case, the nanopillars with a height of 270 nm show the same trend of multipolar resonances with that of *p*=3**r* (Figure 2d), but exhibit slightly reduced transmission as shown in Fig. S3a. The required phase profiles of a single-focal metalens with a focal length *f* follows a hyperbolic function, which is given by

$$\varphi(x,y)-\varphi(o)=\frac{2\pi}{\lambda}\left(\sqrt{x^2+y^2+f^2}-f\right) \qquad \text{Eq. (1)}$$

where *f* is the focal length, λ is the wavelength of the incident light, $\varphi(x,y)$ is required phase at

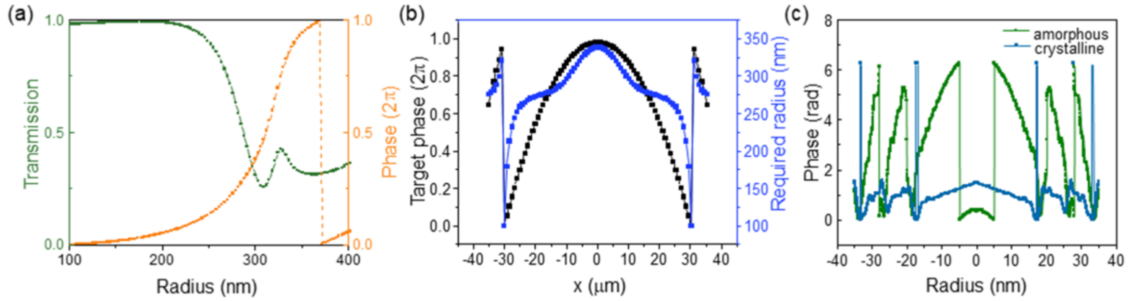

Figure S3. (a) The simulated transmission and phase response for amorphous-state Sb_2Se_3 nanopillars with radius (*r*) gradually increasing from 100 nm to 408 nm. *h*=270 nm, *p*=1020 nm. (b) The target phase calculated from Eq.(1) and the required nanopillar radius. (c) The generated phase modulation when light passes through the metalens when the Sb_2Se_3 is in the amorphous state (green line) and crystalline state (blue line). The phase modulation matches well with the calculated hyperbolic phase profiles with Eq. (1) when the Sb_2Se_3 is in the amorphous state, but mismatches when the materials is in the crystalline state.

any position (x, y) on the plane of the metalens and the origin o is the center of the metalens.[3] Using this equation and the phase-radius relation, we calculate the metalens with varying radii (r) that correspond to the required phase distributions $\varphi(x, y)$ at the designated positions (x, y) as shown in Fig. S3b. Fig. S3c shows the generated phase modulation when light passes through the metalens consisting of nanopillars arranged with the designed radius when the Sb_2Se_3 is in the amorphous state (green line) and crystalline state (blue line). The phase modulation matches well with the calculated hyperbolic phase profiles when the Sb_2Se_3 is in the amorphous state, but mismatches when the materials is at crystalline state.

4. Simulation of intensity-tunable metalens using crystalline-state Sb_2Se_3

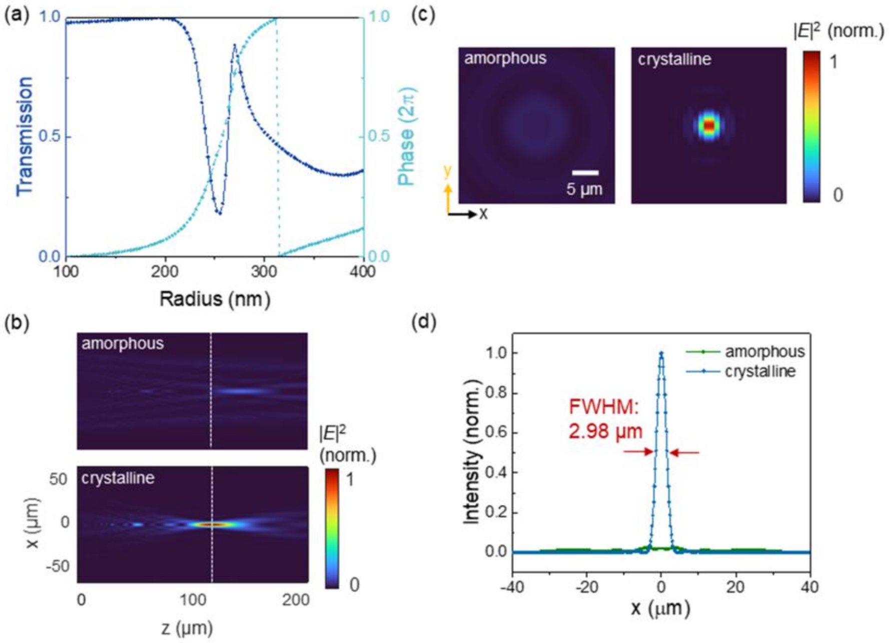

Figure S4. Tunable single-focal metalenses using crystalline-state Sb_2Se_3. The simulated transmission and phase response for crystalline-state Sb_2Se_3 nanopillars with radius (r) gradually increasing from 100 nm to 408 nm. h=225 nm, p=1020 nm. (b) The simulated light intensity distributions at xz-plane when light propagates through the metalens when the material is in the amorphous state (upper) and crystalline state (bottom). (c) The simulated light intensity distributions at the focal plane (z=117.13 μm). (d) The light intensity along x-axis at the focal plane (z=117.13 μm, y=0).

5. The transmission, reflection and absorption of the optimized amorphous-state Sb_2Se_3 nanopillar at the

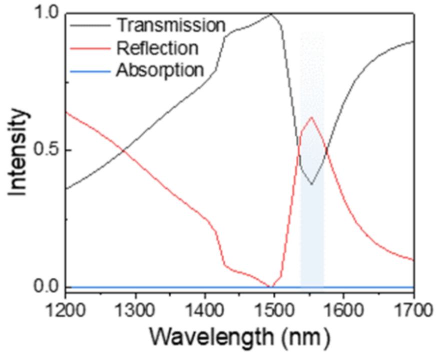

Figure S5. The simulated transmission, reflection and absorption at resonant wavelength (1550 nm) with overlapped ED and MD when light propagates though the amorphous-state Sb_2Se_3 nanopillar with radius of 310 nm and height of 270 nm.

resonant wavelength

6. Near-unity transmission and 2π phase modulation by embedding the nanopillars in an optimized dielectric top layer

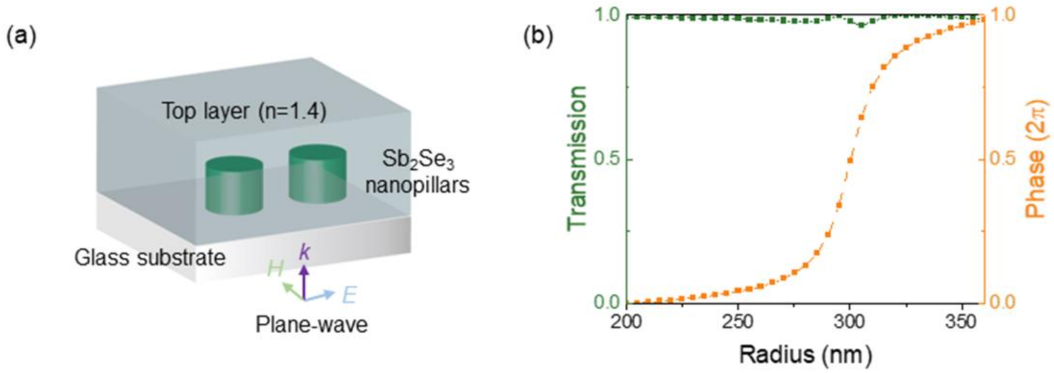

Figure S6. (a) Sketch of the nanopillars embedded in a top layer with a refractive index n= 1.4. (b) The obtained near-unity transmission and 2π phase modulation when the nanopillar radius changes from 100 nm to 360 nm.

Transmission intensity (i.e. focusing efficiency) of a metalens is determined by the mode overlap between electric- and magnetic-dipoles of a Sb_2Se_3 nanopillar. Although Sb_2Se_3 nanopillars show tight field confinement, the difference in coupling strength between two dipoles can still exist due to non-trivial spatial mode mismatch. This can be further improved by either optimizing nanopillar geometries[4] or changing the surrounding environment to higher index dielectrics[5], such as transparent polymer (n ~ 1.4).

By using such techniques, we show the significant improvement of nanopillar transmission with achieving 2π modulation in Fig. S6. This is due to the enhanced electric field confinement within

the nanopillar by replacing surrounding medium with higher refractive index material. However, it is worth mentioning that further numerical optimization is still necessary to avoid or minimize the interelement electromagnetic coupling between the neighboring nanopillars of a metalens in order to improve its focusing efficiency. [6].

7. Schematic of the fabrication process and the measurement setup

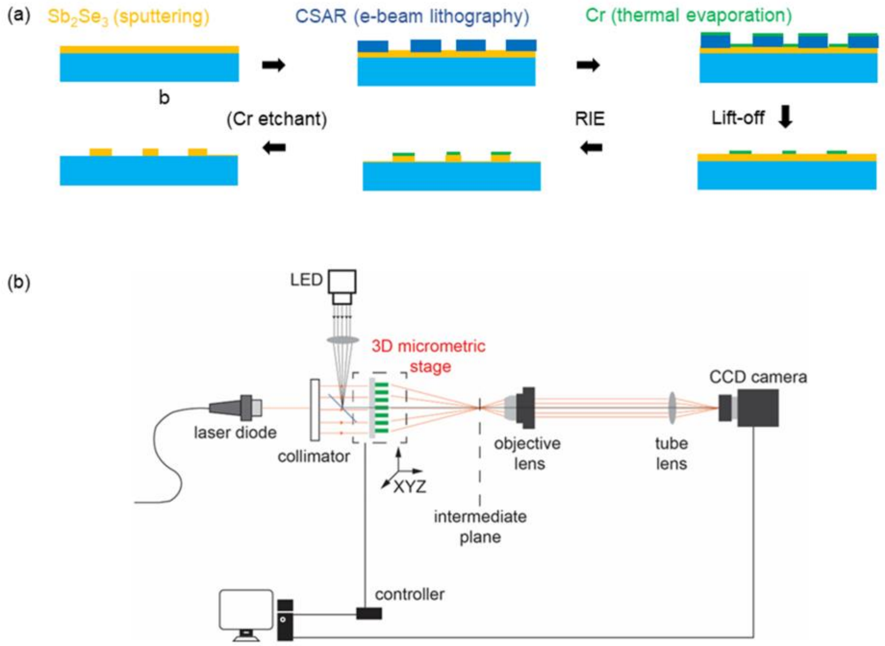

Figure S7. (a) Schematic view of the fabrication process and (b) Illustration of the measurement setup for focusing tests.

Note that in our case, the Cr hard mark is kept at the end as a protection capping layer of the Sb_2Se_3.

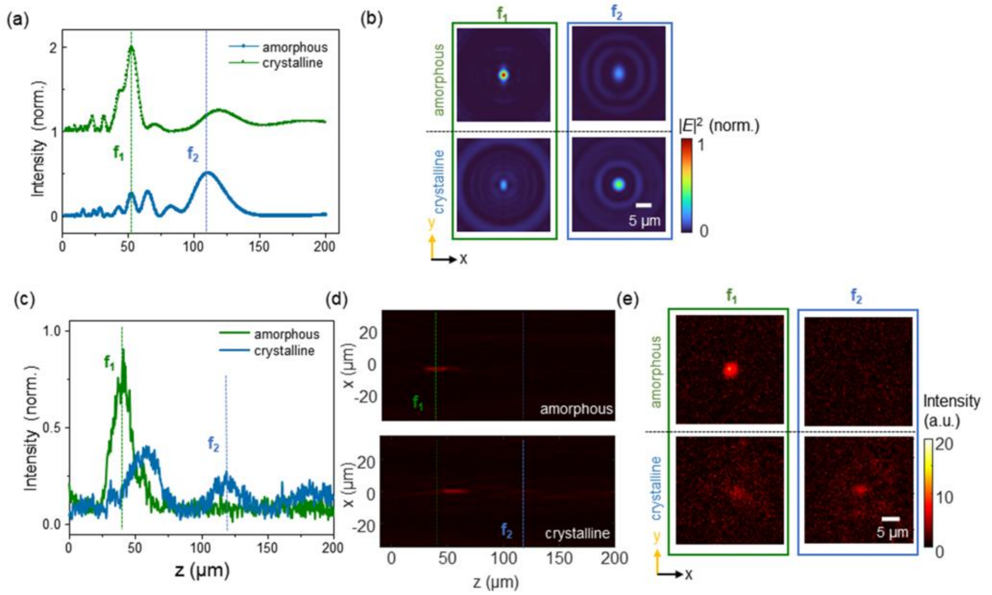

Figure S8. (a) The simulated intensity along z-axis when light propagates through the varifocal metalens. (b) The simulated light intensity distributions at focal plane (f_1: z=52.62 μm, f_2: z=110.18 μm). (c) The measured intensity profiles in the z-direction for amorphous and crystalline-state Sb_2Se_3. (d) The measured light intensity distributions at xz-plane, which is normalized to the maximum intensity at focal point f_1. (e) The measured light intensity distribution at the focal planes.

8. Light intensity distribution along the light propagation direction of the varifocal metalens

9. Focusing performance of each region of the varifocal metalens as a separate intensity-tunable metalens

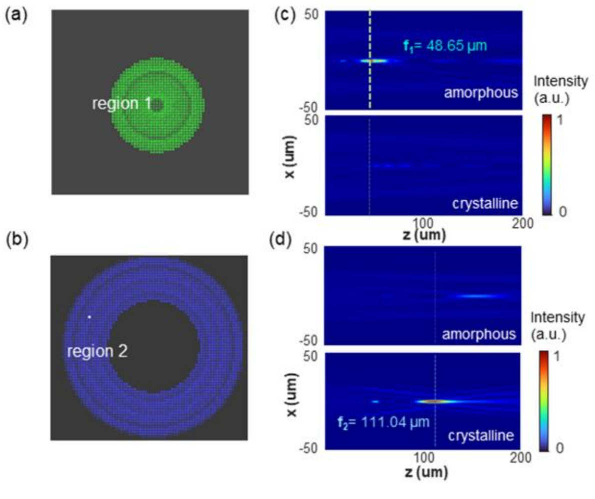

Figure S9. The layouts of simulation structure of region 1 (a) and region 2 (b) and the light-intensity distributions along z-axis of region 1 (c) and region 2 (d), suggesting that both regions function well as intensity tunable metalenses in response to the crystalline state of the Sb_2Se_3.

We calculated the focusing performance of each region separately in Fig. S9. We observed that

the focusing performance of the round-hole metalens (i.e. region 2) is similar to that of the full-circle one in region 1 in terms of the intensity modulation. The round-hole metalens shows the intensity modulation ($\Delta I/I_0$) of 95.07%, while the one for the full-circle one is 96.1%. The FWHM of point-spread-function at focal point for the round-hole metalens is 2.46 μm, while the one for the full-circle one is 2.45 μm. This is because the large feature sizes of region 2 compensates for the degradation of focusing quality that can be caused by the void (i.e. open area). Therefore, each region of the metalens operates as an independent, intensity-tunable metalens with different focal lengths, realizing a varifocal metalens.

10. Broadband performance of the metalens

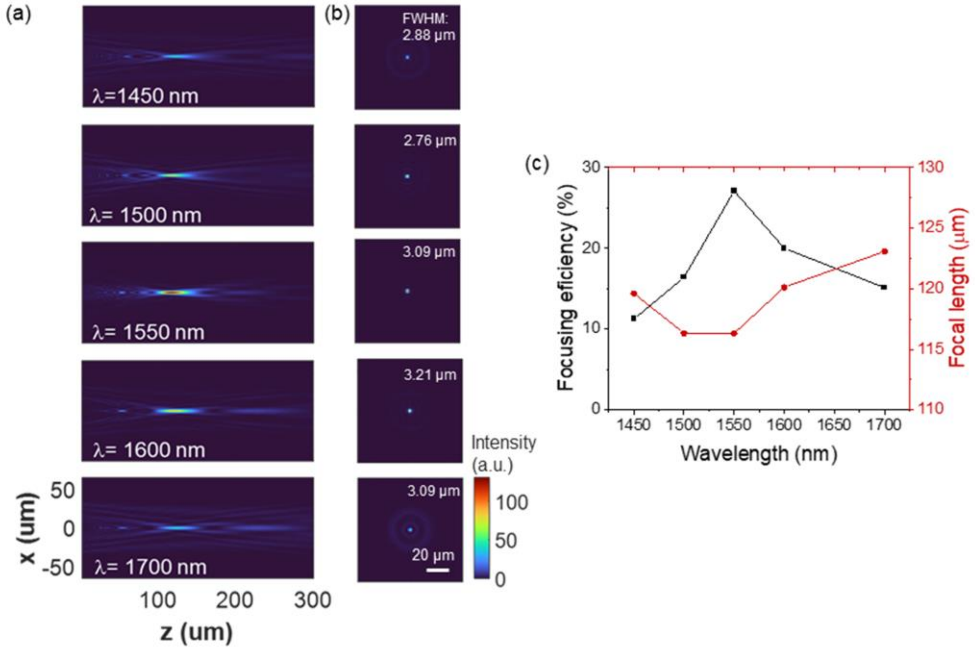

Figure S10. Broadband property of the proposed a-Sb_2Se_3 metalens. (a) The simulated far-filed intensity along the direction of light propagation (left column) and at the focal plane (b) at wavelengths of 1450 nm~1700 nm. (c) Focusing efficiencies and focal lengths as a function of operating wavelength. (d) The relevant phase response of nanopillars with radii from 100 nm to 400 nm for a broadband wavelength range.

As shown in Fig. S10, we calculated the field distribution of a single focal metalens under the illumination at different wavelengths (1450 – 1700 nm). One can observe that the focusing efficiency remains above 15% within the wavelength range between ~1500 and ~1700 nm, while the full-width-half-maxima (FWHM) of their point spread functions at focal point and focal lengths are unaffected (≤10% for FWHM, ≤5.81% for focal length). Therefore, the working bandwidth is estimated to be ~200 nm for both single and varifocal lenses since a varifocal lens is considered as a combination of two single focal lenses.

Table S1. Comparison of the state of the art of varifocal metalenses.

Switching mechanism	Operating wavelength	Polarization-insensitive	Focal length	Efficiency, %	Diffraction-limited focusing	Low-loss material
Phase change in Sb_2Se_3 (single-layer metasurface) This work	1550 nm	Yes	41 μm /123 μm	4.41/3.83	Y	Yes
Phase change in GSST[7] (two-layer metasurfaces)	5.2 μm	No	1.5 mm /2 mm	23/21	Y	Yes
Plasmonic antenna (Au) on thin film of GST phase change material[8]	3.1 μm	No	0.5 mm /1 mm	5/10	N	No
Modulation of light polarization (LC + static Si metasurfaces)[9]	633 nm	No	7.5 mm /3.7 mm	43.5/44.0	Y	N/A
Mechanical movement (MEMS) [10]	915 nm	Yes	565 μm ~ 629 μm	40~45	Y	N/A
Actuation strain (electrical)[11]	1550 nm	Yes	50 mm ~ 65 mm	62.5% (average)	Y	N/A

References

[1] I. Staude *et al.*, "Tailoring directional scattering through magnetic and electric resonances in subwavelength silicon nanodisks," *ACS nano,* vol. 7, no. 9, pp. 7824-7832, 2013.

[2] D. Tzarouchis and A. Sihvola, "Light scattering by a dielectric sphere: Perspectives on the Mie resonances," *J Applied Sciences,* vol. 8, no. 2, p. 184, 2018.

[3] X. Jiang *et al.*, "All-dielectric metalens for terahertz wave imaging," *Optics Express,* vol. 26, no. 11, pp. 14132-14142, 2018.

[4] B. S. Luk'yanchuk, N. V. Voshchinnikov, R. Paniagua-Domínguez, and A. I. Kuznetsov, "Optimum forward light scattering by spherical and spheroidal dielectric nanoparticles with high refractive index," *J ACS Photonics,* vol. 2, no. 7, pp. 993-999, 2015.

[5] M. Decker *et al.*, "High - efficiency dielectric Huygens' surfaces," *Advanced Optical Materials,* vol. 3, no. 6, pp. 813-820, 2015.

[6] C. Gigli, Q. Li, P. Chavel, G. Leo, M. L. Brongersma, and P. Lalanne, "Fundamental limitations of Huygens' metasurfaces for optical beam shaping," *Laser Photonics Reviews,* vol. 15, no. 8, p. 2000448, 2021.

[7] M. Y. Shalaginov *et al.*, "Reconfigurable all-dielectric metalens with diffraction-limited performance," *Nature communications,* vol. 12, no. 1, pp. 1-8, 2021.

[8] X. Yin *et al.*, "Beam switching and bifocal zoom lensing using active plasmonic metasurfaces," *Light: Science & Applications,* vol. 6, no. 7, pp. e17016-e17016, 2017.

[9] T. Badloe, I. Kim, Y. Kim, J. Kim, and J. Rho, "Electrically Tunable Bifocal Metalens with Diffraction - Limited Focusing and Imaging at Visible Wavelengths," *Advanced Science,* vol. 8, no. 21, p. 2102646, 2021.

[10] E. Arbabi, A. Arbabi, S. M. Kamali, Y. Horie, M. Faraji-Dana, and A. Faraon, "MEMS-tunable dielectric metasurface lens," *Nature communications,* vol. 9, no. 1, pp. 1-9, 2018.

[11] A. She, S. Zhang, S. Shian, D. R. Clarke, and F. Capasso, "Adaptive metalenses with simultaneous electrical control of focal length, astigmatism, and shift," *Science advances,* vol. 4, no. 2, p. eaap9957, 2018.
